# VOLCON: a randomized controlled trial investigating complications and functional outcome of volar plating vs casting of unstable distal radius fractures in patients older than 65 years

**DOI:** 10.1186/s10195-022-00673-4

**Published:** 2022-11-28

**Authors:** Rikke Thorninger, Daniel Wæver, Michael Tjørnild, Martin Lind, Jan Duedal Rölfing

**Affiliations:** 1 Department of Orthopaedics, Regional Hospital Randers, Skovlyvej 15, 8930 Randers, Denmark; 2 Department of Clinical Medicine, HEALTH, Aarhus University, Palle Juul-Jensens Boulevard 82, 8200 Aarhus, Denmark; 3 Department of Orthopaedics, Aarhus University Hospital, Palle Juul-Jensens Boulevard 99, J801, 8200 Aarhus, Denmark

**Keywords:** distal radius fracture, conservative treatment, operative treatment, complications, functional outcome PROM

## Abstract

**Background:**

Primary aim: to compare complications of operative vs non-operative treatment of unstable distal radius fractures (DRF) fulfilling national clinical guidelines for operative treatment. Secondary aim: to compare the functional outcomes.

**Materials and methods:**

A single-centre randomized controlled trial of unstable DRF. 50 patients: volar locking plate, 2 weeks casting + 3 weeks orthosis. 50 patients: 5 weeks casting. Primary outcome: complications assessed after 2 and 5 weeks and 6 and 12 months. Secondary outcomes: Quick-DASH, PRWHE, range of motion, grip strength, EQ-5D-3L.

**Results:**

148 patients were screened from November 2019 to March 2021. 48 patients did not want to participate or were unable to participate in the follow-up. 100 patients were randomized and 85 patients were available for full analysis due to there being 4 deaths, 6 withdrawals, 1 wrong inclusion, 1 emigration, 1 refracture, 1 patient with compartment syndrome, and 1 who was advised to undergo surgery after being randomized to non-operative treatment. Median age was 74 years (range 65–92), 81 women/19 men, 42 right/58 left side, 87 retired, 11 smokers, 86 ASA class 1 or 2. Complication rates did not statistical significantly vary between the operative and non-operative group: 20.9% (9/43) vs 16.6% (7/42), *p* = 0.78 (Fisher’s exact test). Complications were driven by sensory disturbances. Four reoperations were performed: two in the non-operative group: carpal tunnel syndrome; two in the operative group: one carpal tunnel syndrome, one protruding screw causing extensor tendon irritation. Mean difference in Quick-DASH varied from 2.3 (95% CI − 3 to 8) pre-injury to 4.2 (− 4 to 12) at 12 months. Quick-DASH and PRWHE were neither statistically nor clinically-relevant different between groups.

**Conclusions:**

Complication rates after operative and non-operative treatment of DRF were similar. Volar plating did not improve the functional outcome after 5 weeks, 6 months, and 12 months. These findings are in line with recent RCTs and mandate a revision of guidelines towards more conservative treatment. Take home messages: (1) consider non-operative treatment in elderly patients sustaining unstable DRFs; (2) choosing operative treatment in patients older than 65 years should not be the gold standard; (3) however, non-operative treatment still carries a risk for complications.

**Level of evidence:**

II.

*Trial registration* Clinicaltrials.gov NCT03716661, registered 23rd Oct 2018; Published protocol PMC6599306.

## Background

Distal radius fractures (DRF) account for 18% of all fractures in the elderly ≥ 65 years of age [1].

The incidence rate of DRF is approximately 190–200 per 100,000 person years and likely to increase in the future [1, 2]. Operative treatment with open reduction and internal fixation (ORIF) using volar locking plates is the recommended standard treatment of unstable DRF according to the National Clinical Guidelines (NCG) [3] issued by the Danish Health Authorities, similar to the American Academy of Orthopedic Surgeons [4].

The NCG recommend operative treatment of DRF that fulfils the following radiologic criteria after attempted closed reduction:

-  > 10° dorsal tilt of the radius
-  > 2 mm articular step-off
-  > 3 mm ulnar variance
- Incongruence of the distal radioulnar joint,
- Substantial dorsal comminution indicating gross instability.

If one or more of these criteria are met, the advice is to use ORIF most often with a volar locking plate regardless of the patient’s age.

However, according to the NCG, the scientific evidence is “very weak” for this recommendation compared to that of closed reduction and cast immobilization. Nonetheless, in 2021, the vast majority of unstable DRF were treated by volar plating according to guidelines. Furthermore, volar plating surgery is associated with a significant complication rate of up to 30% [5, 6].

Recent evidence indicates that non-operative treatment may deserve the role of gold standard in the elderly population [7–10].

This randomized controlled trial (RCT) aims to compare unstable DRF treated with plaster cast immobilization for 5 weeks with ORIF with a volar locking plate in terms of complication rate, functional outcome and patient-reported outcome in patients ≥ 65 years.

We hypothesized that treatment of unstable DRF with non-operative treatment would be superior to ORIF with a volar locking plate in terms of complication rate. However, both treatments are expected to have comparable functional and patient-reported outcomes after 12 months (mean difference in QuickDASH < 16).

## Materials and methods

We conducted a prospective, single-centre, assessor-blinded, randomized, controlled superiority trial comparing non-operative treatment (*n*1 = 50) vs volar plating (*n*2 = 50) of unstable DRF in patients ≥ 65 years with regards to complications and functional outcome. The detailed study protocol has been published with open access [11]. The study was conducted from November 2019 to March 2022.

### Interventions and randomization

**Fig. 1:**
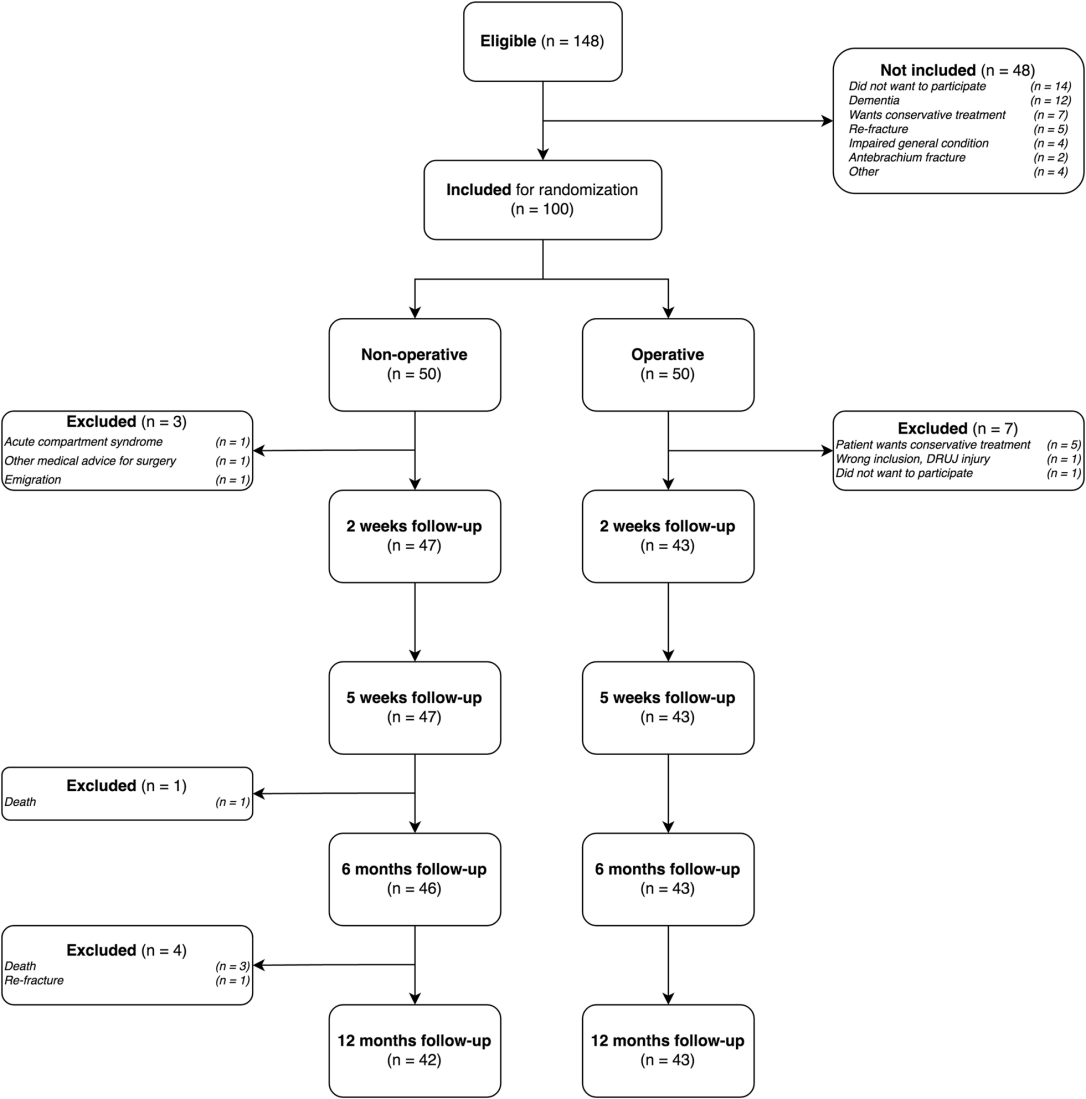
Consort flowchart

All patients with DRF diagnosed at our emergency department (ED) were screened for eligibility. Exclusion criteria were age < 65 years, high energy fracture, open fracture, concomitant injuries, previous fracture on the same arm, and inability to give written consent (Fig. 1).

After diagnosing the DRF on standardized wrist radiographs (anterior–posterior and lateral projections) in the ED, the physician on call had two attempts to achieve an acceptable closed reduction under local analgesia with a 20 mg/ml lidocaine haematoma block. The radiological NCG criteria were assessed on new radiographs. According to the sample size calculation, 50 participants were allocated to each group; hence, 100 identical A5 envelopes were sealed, each containing a folded note whereupon either “operative” or “non-operative” were written. The concealment strategy was tested and light could not shine through the envelopes. Concealment of allocation was thus effective.

ORIF with volar plate fixation utilized Acu-Loc^®^, Acumed or Variax^®^, Stryker, depending on the surgeon’s preference. A standard Henry approach to the distal radius and pronator quadratus repair, if possible, was performed in the operative group. The vast majority of patients were operated under regional anaesthesia, and the remaining patients were operated under general anaesthesia.

After surgery, the wrist was immobilized in a dorsal plaster cast for 2 weeks, followed by a further 3 weeks of immobilization with a removable orthosis. A single hand therapeutic instruction took place.

Non-operative treatment consisted of a dorsal plaster cast immobilization for 5 weeks. Only discomfort, neurologic deficits or signs of infection warranted removal and replacement with another dorsal plaster cast. A single hand therapeutic instruction took place in this group after removal of the cast. No radiological evaluation was performed before 5 weeks after the injury.

### Outcomes

The primary outcome was the complication rate after 12 months. Complications were prospectively recorded at day 0 (baseline), 2 weeks, 5 weeks, 6 months and 12 months after injury. The patient answered standardized questions from the investigators at the given timepoints.

Complications were defined as the presence of:

- Sensory disturbance, including carpal tunnel syndrome and chronic regional pain syndrome
- Flexor tendon rupture and irritation
- Extensor tendon rupture and irritation
- Hardware failure, e.g. osteosynthesis loosening
- Infection: superficial (treated with antibiotics only) or deep (requiring surgical intervention)
- Reoperation with hardware replacement
- Reoperation with hardware removal (partial or total), which is not routinely performed in our country
- Vascular compromise (capillary refill ≥ 2 s).

Secondary outcomes were obtained at the same timepoints as the primary outcomes.

Patient-reported outcome measures included the Danish version of the Quick Disabilities of the Arm, Shoulder and Hand (Quick-DASH), which was used to assess the level of functionality prior to injury and after 2 weeks, 5 weeks, 6 months and 12 months. The minimal clinically relevant difference was defined as a 16- to 20-point difference in Quick-DASH [12–14]. The pain experienced during activity within the preceding 14 days before the injury and at 2 weeks, 5 weeks, 6 and 12 months of follow-up was recorded using the 0–10 Numeric Rating Scale (NRS). A validated Danish version of the Patient-Rated Wrist/Hand Evaluation (PRWHE) was also applied [15].

Range of motion (ROM) was measured by a registered nurse using a goniometer. To ensure the observer was blinded, the patient was instructed not to talk about the treatment. Furthermore, all wrists were covered by a glove concealing potential scars.

The grip strengths of both left and right hand were estimated as the mean score of three repetitions of each hand, alternating hands between attempts, after 6 months and 12 months using a calibrated dynamometer (EH101 CAMRY, by the Camry scale). Quality of life was assessed with European Quality of Life 5 Dimensions 3 Levels (EQ-5D-3L). 

Baseline demographics were reported as age, gender, side of DRF, dominant hand, working status, ASA class 1–6 (American Society of Anaesthesiologists Classification), smoking, alcohol consumption and diabetes.

### Statistical methods

The primary outcome, complication rate, was compared using Fisher’s exact test of the accumulated complication rate after 12 months. In order to prevent double counting, in patients with multiple complications, only one complication was accounted for in this calculation (bold numbers in Table 2).

All secondary outcome measures were analysed for all obtained data using mixed-effects analysis with Sidak’s multiple comparisons test. All available data were used without imputations for missing values.

According to our sample size calculation, 50 patients per treatment arm provide 80% statistical power at a 5% alpha level assuming a difference of 20% in complication rate between operatively and conservatively treated patients.

Statistical analyses were performed with Prism 9 for macOS.

The present trial was approved by the Danish Scientific Ethical Committee (ID: 1-10-72-420-17) and registered at Clinicaltrials.gov (ID: NCT03716661) [7].

## Results

A total of 148 patients were screened for eligibility between November 2019 and March 2021. Due to the COVID-19 pandemic and the lower incidence of fractures during the pandemic, the inclusion period was longer than expected [16–18].

Of those 148 patients, 48 were excluded mainly because they did not want to participate or were excluded due to the stipulated exclusion criteria. 100 patients were randomized to either operative or non-operative treatment. A total of 85 patients were available for complete data analysis after 12 months. All patients stayed in their randomized group and none were allowed to cross over (Fig. 1).

Baseline demographics are given in Table 1. The vast majority of patients in both groups were healthy, active retired individuals with low ASA class scores.

**Table 1:** Baseline demographics

	Non-operative (n=50)	Operative (n=50)
Female / Male	40 (80%) / 10 (20%)	41 (82%) / 9 (18%)
Median age (min., IQR, max.) [years]	74 (65, 69-82, 91)	75 (65, 70-80, 92)
Fractured side: R / L	24 (48%) / 26 (52%)	18 (36%) / 32 (64%)
Hand dominance: R / L / Ambidextrous / Missing data	46 (92%) / 1 (2%) / 1 (2%) / 2 (4%)	38 (76%) / 3 (6%) / 0 (0%) / 9 (18%)
Dominant side fractured	23 (46%)	16 (32%)
Retired / Working / Volunteer / Retired / Missing data	45 (90%) / 1 (2%) / 2 (4%) / 2 (4%)	42 (84%) / 0 (0%) / 0 (0%) / 8 (16%)
Smoking: Yes / No / Missing data	9 (18%) / 37 (74%) / 4 (8%)	2 (4%) / 36 (72%) / 12 (24%)
Alcohol overconsumption: Yes / No / missing data	8 (16%) / 38 (76%) / 4 (8%)	6 (12%) / 32 (64%) / 12 (24%)
ASA class 1 / 2	13 (26%) / 30 (60%)	14 (28%) / 29 (58%)
ASA class 3 / 4-6 / missing data	6 (12%) / 0 (0%) / 1 (2%)	4 (8%) / 0 (0%) / 3 (6%)
Comorbidities:		
Hypertension	23 (46%)	16 (32%)
Diabetes	6 (12%)	2 (4%)
Depression	4 (8%)	1 (2%)
Osteoporosis	3 (6%)	3 (6%)
Prescribed medications: None / 1-4 / ≥ 5	8 (16%) / 26 (52%) / 16 (32%)	19 (38%) / 21 (42%) / 10 (20%)

R = Right, L = Left

A fracture in an ambidextrous patient was not considered a fracture of the dominant side.

Alcohol overconsumption was defined as more than 7 units/week for females and 14 units/week for males.

### Primary outcomes

The primary outcome complication rate after 12 months was 16.6% (7/42) in the non-operative group and 20.9% (9/43) in the operative group (*p* = 0.78, Fisher’s exact test, Table 2). Patients with multiple complications were only accounted for once.

**Table 2:**
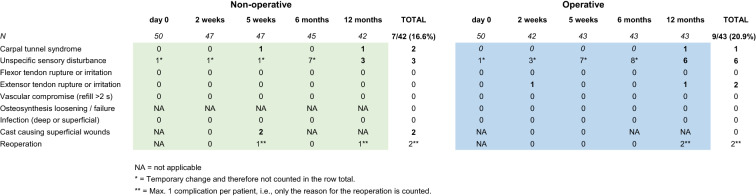
Complications

Many patients reported sensory disturbances at given time points; however, most often, these disappeared in follow-up visits. Consequently, only sensory disturbances at the 12-months visit contributed to the stated complication rate. Furthermore, sensory disturbances were not nerve specific. However, some carpal tunnel syndromes were registered. We did not observe any complex regional pain syndromes.

In 42 non-operatively treated patients, the complication rate was 16.6% due to 7 events: 2 carpal tunnel syndromes causing "reoperations", i.e., median nerve decompression after 5 weeks and 12 months; 3 unspecific sensory disturbances at 12-months follow-up; and 2 superficial wounds without infection at cast removal after 5 weeks.

**Fig. 2:**
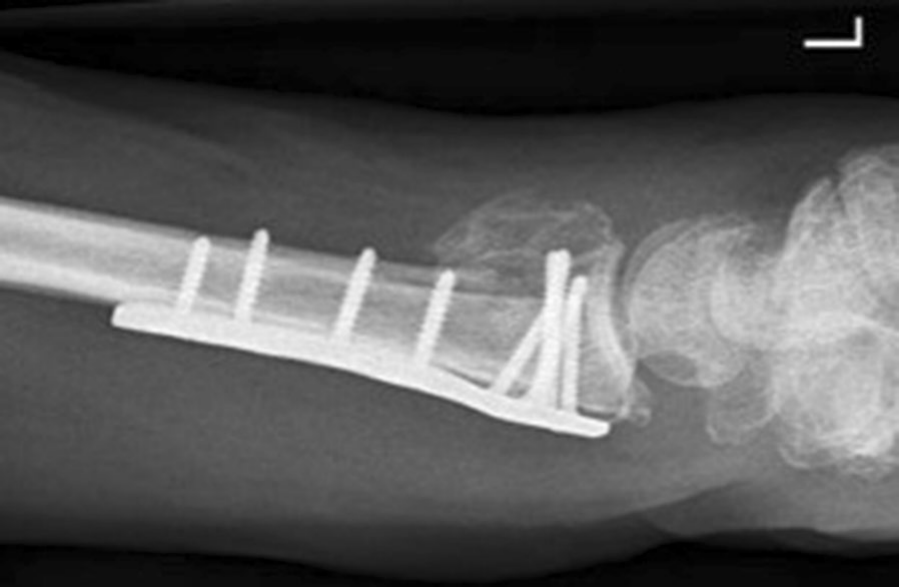
Bent volar locking plate. Lateral wrist radiograph of an operatively treated distal radius fracture patient after a new fall causing a re-fracture and bending of the volar locking plate. This was a new trauma and therefore not accounted for as a complication

In 43 operatively treated patients, the complication rate was 20.9% due to 9 events: 1 carpal tunnel syndrome causing a reoperation, i.e., plate removal and nerve decompression after 11 months; 6 unspecific sensory disturbances at 12 months follow-up; 1 extensor tendon irritation due to a protruding screw that caused plate removal after 12 months; and 1 extensor pollicis longus rupture that was not repaired. Thus, two reoperations were performed in the operative group. Another patient in the operated group fell again and sustained a new DRF and bending of the volar plate (Fig. 2). This new trauma was not accounted for as a complication/reoperation. Lastly, 3 trigger fingers after 5 weeks, 6 months, and 12 months were observed in the operative group and none were observed in the non-operative group. However, these were not classified as complications.

Furthermore, in the non-operative group, one cast was changed due to loosening after the initial swelling had subsided, and one patient complained about ulnar wrist pain at final follow-up. In the operative group, three patients complained about a bothersome decrease in ROM. These events/complaints were not accounted for as complications, but were disclosed in order to report all data.

### Secondary outcomes

**Fig. 3:**
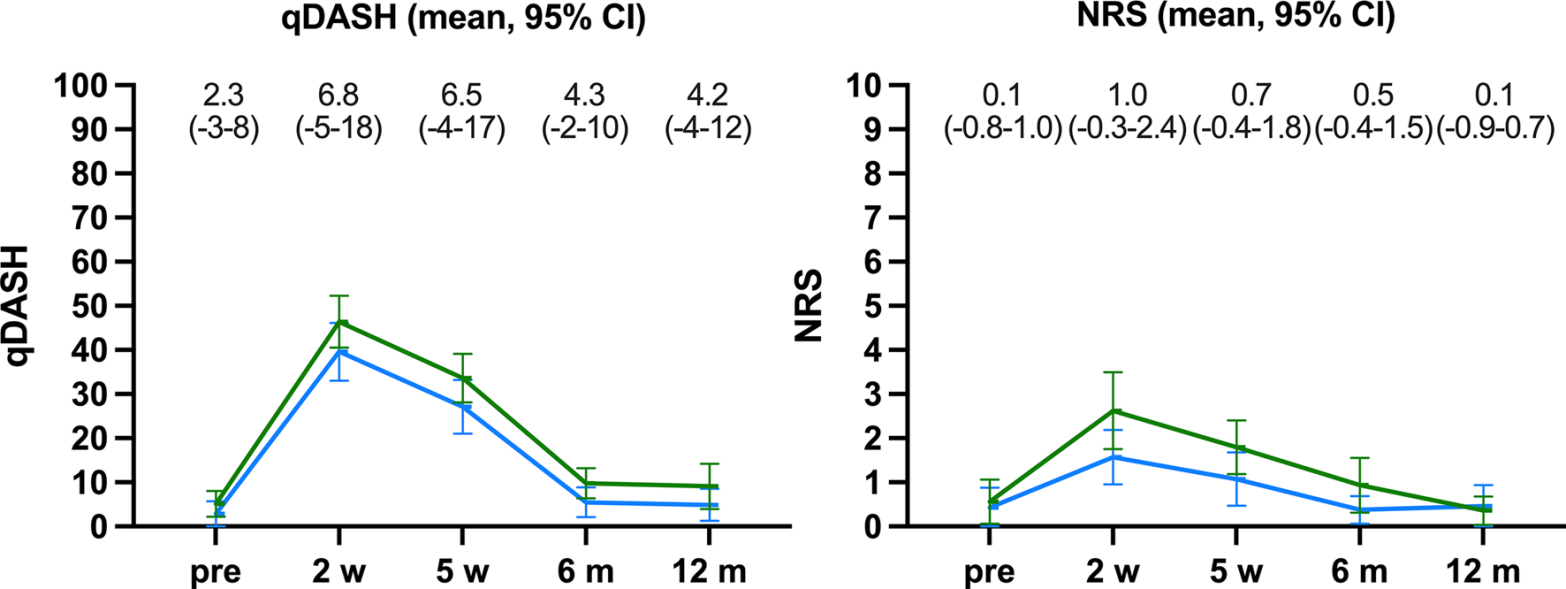
Functional outcome. Mean qDASH and NRS with 95% confidence intervals as error bars are depicted pre-injury (*pre*) and after 2 and 5 weeks (*w*) and 6 and 12 months (*m*). The mean difference (95% confidence interval) between the operative group (*blue*) and the non-operative group (*green*) is given above the timepoints

According to Fig. 3, Quick-DASH and NRS did not statistically significantly differ between the operative and non-operative group at any timepoint. Furthermore, after 6 months and 12 months there was no statistically significant difference compared with the recalled pre-injury state in either group.

Mean PRWHE was similar in the operative and non-operative groups after 6 months: 9.6 (4.5–14.7) vs. 12.6 (8.7–16.5) and after 12 months: 8.6 (2.5–14.7) vs. 8.0 (3.6–12.4). There was no statistically significant difference between the two different treatments at any timepoint, but there was a slight overall improvement from 6 to 12 months: mean difference 2.8 (0.1–5.5), *p* = 0.04.

The active ROM improved throughout the observation period of 12 months. Many movements were statistically significantly reduced compared with the healthy side after 5 weeks and 6 months, but none was statistically significantly different from the healthy side after 12 months. Comparisons between the two treatment groups revealed a statistically significant difference in combined flexion–extension ROM after 5 weeks: mean diff. 14.7° (5.5–23.8°, *p* < 0.0001) and 6 months: mean diff. 9.8° (0.3–19.3°, *p* = 0.037). The mean difference after 12 months was 6.8° (− 3.2 to 16.7°,* p *= 0.61). At all time points, flexion–extension ROM slightly favoured the operative group.

The grip strength after 12 months was: mean (injured side, operative): 16.2 (14.0–18.5) kg; mean (uninjured side, operative): 17.1 (14.9–19.4); mean (injured side, non-operative): 14.8 (12.5–17.1); and mean (uninjured side, non-operative): 18.1 (15.0–21.3) kg. At 12 months follow-up, the mean difference in grip strength was 1.4 (− 2.6 to 5.5) kg. Mixed model analysis of the grip strength revealed a significant time effect (*p* < 0.0001), but no treatment effect (*p* = 0.23). Median EQ-5D-3L indices did not statistically significantly differ between the operative and non-operative group after 12 months (1.0 (range 0.36-1.0) vs. 1.0 (range 0.14-1.0)).

## Discussion

The most important finding of the present study was that there was no statistically significant difference in complication rate or in functional or patient-reported outcome measures between operatively or non-operatively treated unstable DRF in patients ≥ 65 years after 12 months.

The similar complication rates between the operative and non-operative groups are supported by meta-analyses combining RCTs on non-operatively versus operatively managed DRFs [5, 7, 9, 10, 19–21]. Our results regarding complication rates after DRF are thus in line with previous studies on the subject. However, the definition of complications varies in the literature, and these results are therefore challenging to interpret. One meta-analysis divided complications into minor and major and found a significant higher rate of major complications in the operative group [21]. We report complications adhering to the published protocol and therefore did not subdivided them into major and minor complications [11]. Moreover, we observed 3 trigger fingers in the operative group and none in the non-operative group. However, these observations were not counted as complications, because these events were associated with operative treatment but were not necessarily caused by it.

Regarding patient-reported outcome measures, i.e., the Quick-DASH score, similar results for operatively and non-operatively treated patients were reported in the existing literature [10, 20]. However, a meta-analysis that included not only RCTs but also prospective studies found a significantly lower Quick-DASH score favouring the operative group in the first year [19]. That meta-analysis found an effect size of − 5.22 (95% CI − 8.87 to − 1.57). In the present study, the mean difference in Quick-DASH was 4.2 (95% CI – 4 to 12), which was also well below the threshold for a minimal clinically relevant difference, 16–20 points. Likewise, the ROM was similar between groups, and the statistically significant difference in flexion–extension ROM at 5 weeks and 6 months was barely clinically relevant, i.e., the mean difference after 12 months was 6.8° (− 3.2 to 16.7°, *p* = 0.61).

Not all patients are ideal for non-operative treatment. The NCG recommend operative treatment in older patients with unstable DRF unless they have a low functional demand [3]. The same holds true in Norway [22]. In contrast, based on the same literature that was available in 2015, the Finnish Medical Society highlights that there is no difference in functional outcome and therefore recommends non-operative treatment to avoid costs and complications [23]. Furthermore, the latest British guidelines from 2018 state: “In patients 65 years of age or older, non-operative treatment can be considered as a primary treatment for dorsally displaced DRF unless there is significant deformity or neurological compromise” [24, 25]. It should be noted that both the Danish and British guidelines are more than 5 years old, and the majority of RCTs on this topic were published afterwards. These trials call for revisions of the guidelines towards a non-operative approach for the vast majority of patients.

So far, no long-term results of high-quality RCTs have become available. Theoretically, the functional outcome may decline over time in the non-operative group due to early onset of post-traumatic osteoarthritis, stiffness and pain.

The size of the study population is a limitation of our study. Performing the sample size analysis, we estimated complication rates for operative treatment based on our own retrospective complication rate in 576 patients [6]. The observed complication rate in the non-operatively treated group was higher than anticipated in the sample size calculation. Consequently, the power of the present trial was not sufficient to find statistically significant differences. However, considering the results of the meta-analyses discussed above, the present study adds to the evidence that complications are also to be expected in non-operatively treated DRF patients.

Another limitation of the present study is that it was not double blinded. We refrained from sham surgery due to the fact that most DRF patients at our institution are wide awake during surgery. We found it unethical to operate under general anaesthesia in order to ensure proper blinding of the patients. Lawson et al. state that people are more likely to rate their treatment as successful when they have had surgery [9]. However, we did perform assessor blinding of three trained nurses as described above and depicted in the published protocol [11].

Performing a single-centre RCT with a relatively small research group ensured a high level of control and consistency. All patients potentially eligible for inclusion were assessed by the same consultant and the risk of selection bias was minimized. Data collection was also performed by only a few persons, ensuring uniform data collection.

## Conclusions

Complication rates after operative and non-operative treatment of DRF were similar. Volar plating did not improve the functional outcome after 5 weeks, 6 months, and 12 months. These findings are in line with recent RCTs and mandate a revision of guidelines towards more conservative treatment. Take home messages: (1) consider non-operative treatment in elderly patients sustaining unstable DRFs; (2) choosing operative treatment in patients older than 65 years should not be the gold standard; (3) however, non-operative treatment still carries a risk for complications.

## Abbreviations

DRF: Distal radius fractures
ED: Emergency department
EQ-5D-3L: European Quality of Life 5 dimensions 3 Levels
NCG: National Clinical Guidelines
ORIF: Open reduction and internal fixation
PRWHE: Patient-Rated Wrist Hand Evaluation
Quick-DASH: Quick Disabilities of the Arm, Shoulder and Hand
RCT: Randomized controlled trial
